# The Induction of Tumours of the Subcutaneous Tissues, Liver and Intestine in the Mouse by Certain Dyestuffs and their Intermediates

**DOI:** 10.1038/bjc.1956.79

**Published:** 1956-12

**Authors:** Georgiana M. Bonser, D. B. Clayson, J. W. Jull

## Abstract

**Images:**


					
653

THE INDUCTION OF TUMOURS OF THE SUBCUTANEOUS TISSUES,

LIVER AND INTESTINE IN THE MOUSE BY CERTAIN DYE-
STUFFS AND THEIR INTERMEDIATES

GEORGIANA M. BONSER, D. B. CLAYSON AND J. W. JIULL
From the Department of Experimental Pathology, and Cancer Research,

School of Medicine, University of Leeds

Received for publication October 16, 1956

THE purpose of the experiments about to be described was to test a number of
amines and azo-dyes for carcinogenic activity in the mouse, a species which
has been comparatively little used for the investigation of this type of compound.
The chemicals chosen were 2 dyes (auramine and magenta), the manufacture of
which constitutes an industrial hazard according to Case and Pearson (1954);
6 dyes formerly or at present in use as food colorants (carmoisine, rhodamine B,
sunset yellow, ponceau 2R, xylylazo-2-naphthol and o-tolylazo-2-naphthol);
3 dyestuffs intermediates (benzidine, 1-naphthylamine and 2-naphthylamine);
and 2 ortho hydroxy amines related to known metabolites of aromatic amines
(3: 3'-dihydroxybenzidine  hydrochloride  and  0-methyl-2-amino-1-naphthol
hydrochloride).

The tumours induced by the chemioals were of three types: subcutaneous
sarcomas, hepatomas and intestinal polyps and carcinomas.

Spontaneous intestinal tumours in the mouse have been described occasionally
(Table I). Murray (1905, 1908) found 2 adenocarcinomas in the small intestine
invading the wall of the gut and expanding under the peritoneum. The drawings
of these tumours clearly indicate their nature. Slye, Holmes and Wells (1917)
described a squamous carcinoma of the rectum arising in the prolapsed organ,
the only intestinal tumour observed in 16,500 post-mortem examinations on mice.
Slye (1924) mentioned that 2 squamous caroinomas of the rectum had been
seen at post-mortem in 40,370 mice and one of the duodenum in 1600 mioe. Strong
(1941) stated that strain A mice were liable to develop spontaneous tumours
of the caecum.

Bonser and Jull (unpublished observation) found two caecal polyps in a female
albino stook mouse, bought from a dealer but having no relation to the mice used
in the present experiments, and treated for 136 weeks by means of subcutaneous
injections of arachis oil. These tumours were composed of irregular intestinal
glands, some of which were cystic and dipped through the muscularis muoosae
into the subepithelial tissues, there evoking a mild inflammatory reaction. They
also observed a caecal carcinoma in a female mouse of the same stook in whioh
a paraffin wax pellet had remained implanted in the bladder for 40 weeks with
negative result. The mouse was thus 52-56 weeks of age. This tumour was an
anaplastic bowel carcinoma, invading the musoular wall as far as the serosa
and permeating subserosal and mesenteric lymphatic vessels. Embedded in the
tumour were cystic spaces lined by mucus-secreting columnar epithelium similar
to those seen in the polyps, but situated at all levels in the muscular wall and also
beneath the serosa. From this appearance it was deoided that the tumour arose
in a benign polyp.

INDUCTION OF MOUSE TUMOURS BY DYESTUFFS

Tumours have been induced in the mouse intestinal tract by a variety of
ohemioals (Table II). Badger, Cook, Hewett, Kennaway, Kennaway, Martin
and Robinson (1940) described a oaroinoma of the small intestine in one of 10
mioe receiving 6-methyl-3: 4-benzphenanthrene by mouth for 394 days. Lorenz
and Stewart (1940) described local and metastasising carcinomas of the small
intestine in mioe of the A strain receiving 1: 2: 5: 6-dibenzanthracene and of the
A baokeross receiving 20-methylcholanthrene in an olive oil emulsion in the
drinking water. Later (1941) these authors found similar tumours in mioe of
other strains treated with these chemicals, together with one oarcinoma of the
caecum and a few in the upper part of the colon. White and Stewart (1942)
observed small intestinal polyps and caroinomas and also haemangio-endotheliomas
in the intestinal wall and other abdominal sites in mice of the C3H and C strains
receiving methylcholanthrene in the diet. The photographs and morbid anatomical
descriptions of these tumours are extremely lucid. More males than females
were treated and only one tumour was stated to have occurred in a female. Foulds
(1947) described a small intestinal carcinoma with a secondary deposit in a regional
lymph node in one male out of 25 receiving 2-acetylaminofluorene in the diet.

Thus it would appear that spontaneous benign and malignant tumours of the
intestine occur but rarely in the mouse. The sex incidence is not known. Induced
tumours resemble the spontaneous ones in site and structure and have been
more commonly described in males.

Source of mice.-The mice were brought from a local dealer, who has now
gone out of business. They were divided into groups of 30, comprising equal
numbers of each sex, and were kept 5 in a box. Where the mortality was high
in the early weeks of the experiment, another 30 mice were subsequently added.
They were fed on rat cake, supplemented by cod liver oil and marmite and were
given water ad lib. All mice used in the experiment subsequent to April 1953
were vaccinated on the tail with calf lymph as a protection against ectromelia.

Administration of the chemicals.-At first, a standard technique was adopted
of subcutaneous injection twice weekly of a solution or suspension of the chemioal

EXPLANATION OF PLATES

FIG. 1.-Mouse H 285, male. Forty-six weeks from start of treatment with benzeneazo-2-

anthrol. Vasoformative sarcoma or haemangio-endothelioma of back muscles. A fragment
of the destroyed vertebral body is seen top middle. x 150.

FIG. 2.-Mouse H 127, male. Seventy weeks from start of treatment with arachis oil. Benign

early polyp of caecum, showing regular proliferating glands lined by tall epithelium with
hyperchromatic nuclei and granular cytoplasm, secreting little mucus, situated superficial
to the muscularis mucosae. There is inflammation and fibrosis of the stroma. x 102.

FIG. 3.-Mouse H 69, female. Sixty-five weeks from start of treatment with o-tolylazo-2-

napthol. Benign polyp of ileum, composed of irregular proliferating glands which are
beginning to invade the muscular coat (top centre). There is fibrosis of the stroma. x 40.
FIG. 4.-Mouse H 702, female. Seventy-six weeks from start of treatment with 0-methyl-2-

amino-l-naphthol. Benign polyp of caecum, with cystic glands penetrating the muscular
coat and provoking a zone of inflammation in the serosa. x 26.

FIG. 5.-Mouse H 379, female. Eighty-four weeks from start of treatment with fresh oily

solution of 2-naphthylamine (B.D.H.). Malignant polyp of caecum, composed of irregular
glands lined by tall or cubical epithelium, with hyperchromatic nuclei, granular cytoplasm,
and little mucus secretion, though some of the glands are cystic. The line of the base is
irregular and the muscularis mucosae is partially destroyed. x 102.

FIG. 6.-Mouse H 97, male. Sixty-two weeks from start of treatment with o-tolylazo-2-

naphthol. Anaplastic carcinoma invading the muscular wall as far as the serosa . Tumour
emboli present in subserosal lymphatic ve?sels. Two cystic glands in muscle resembling
those seen in benign polyps. x 30.

654

BRITISH JOURNAL OF CANCER.

p!!?*?j?&?

1

Nkr ;    --     ..^. S

i_  w   s     w]Z               _~~~~~~~~~~~~th

@ "  B.  t-^      s  :,\~4

T.vz q  W..,'-1tm^n, mXf{Je' 'i

4tt, 1

2

Bonser, Clayson and Jull.

VOl. X, NO. 4.

BRITISH JOURNAL OF CANCER.

3

4

Bonser, Clayson and Jull.

Vol. X, No. 4.

BRITISH JOURNAL OF CANCER.

p ?

fk ? u..A

5

6

Bonser, Clayson and Jull.

Vol. X, N4O. 4.

GEORGIANA M. BONSER, D. B. CLAYSON AND J. W. JULL

0

M      x

r-

00 Cs

C    2

.00

0.0

e- Xe co C:,

0
CC

'4
1.

AI  I

*-  *  0 *

04  ~ *  14   1 0

P-4

vt
0o

II  I  I  I

0~~~~~~~~0
O~        0

co  e  00

Ca i

4)*. . ** a4)1

* ~  .2 *. -H

014
H ~~~~~~~~~~~~~~-

+2  *.         0 .   +

Z       0    0

4      4
uZ   oo ^ m-   2

Ca   45  p

H XS

0

0 01  1    1

'4

I     I

.ZOO 40

Ca       Cso

_ . Q .~

0

0 m  Ca

04 0  ;+2,. -

0

taQ0.4.e

o

a       022Q

.4

. A

4 S .  *  *~'

1..0

e '02:

oro
4).

1.4

00
=00

"114

I   I I   I  I   I  I   I

0

0

1   1  1  1 5  : 1

H

I               I I        I    I

I I I I

=Ao ? X ? co 8

44D0          0

0       *           | | o.  I   * 0

-4.-        0~~~~~~~~~~~~~-
0  a 3  .64 01  5  0
t0,  2  14 SE0,2 0

0  ~~~~~~~~C0

0= Ot.-  x0
Z I2     I      Iz a  ISg e

? I s cr C00e. >   < I
0  A  A

0              00,-

45~~~~~~~~~~1

-~~~~~~~  0 ~ ~ ~ ~ ~ ~ ~ ~ 0

0  0 A0 Q@2

Ca      .0       0

GO

'0  '0~~~~~~~~~0

4
p
t-

4

c
11
I
E
4

c
9

2

p       c
3

-4.;

-6a
c
141

r-

4 2  -a

9 t-

a 2  -4a Lo
z Q mu

~)uQ  /\D /

,'2;

44) "
'.12 -. 0

'    '4  x

Om   I

002 'W p,
oI o    ,a

4)
C)

0

:   I

Qv
c

0

0.

4)
0
'4
(D

655

*W I

4)

.t

0+2

4)14

0 C2
0 0

-z&~

a)1
0

z

w4+

4)14

zGo

z

0

0

4.;

w

.S3 :
aD s

,Z

-. W

I

-4

-      s   - c

GEORGIANA M. BONSER, D. B. CLAYSON AND J. W. JULL

04

Z- z ^

0      t
z       '

o0 0 0           0 oo          o        o
CO  CO   CO  CO     CO CO      CO       C

o                    0
CO                  cO

fn 0~~~~"~
03

0   la 0          0

00  C)

0  03  Ca~ ~ ~~ 2

14~~~~~~~~~~~~~.

0 .  4  `  D   .  .- "  0 4

U)  ~ ~ ~ ~ g s ~ ~ ~P4  )   ~ + C o

0~~~~
0~~~~~~~~~~~~~~~~~~~~~~~~~~~~~4

CO

)      cS-4-

*    b

Cl   Cl

CO CO

b)     Z bbb       bX

1-     -4 1-      1-         Cl

CO    CmmC         CO        C

vo Q Q   w  S   S   55      5     5     55       5

,4 oCO oO         CO   CO  cO    eC    Cl    CO0i     6

O~~~ ~ ~ to                            elQ     C8C
04~~~~~~~~~~~~~~~~~~~~~~~4

(D                P ,0       ;0 0 3O 't rn  02 C)0  C),Q

_+ -4  ?  03
14~~~~~           1~    4     P4    P444    P41

0 (D              -4~C)

0              11~~~~~~4- 0 p   1440  4.40  44.00i

0 *              00  )      o"    0*0   0.0       00

-34~1                              -0            O.,C)

0  0              c5.0P4                O +

~~~0  +4  +4    .~~+4  4  - 4

*  .  .-   .~~~          .wI  +

00       00       P C3       0    0

N       04~~~~~~~~~~~~~~~~~~~~~~~~~~~~~~~~~~Q

.4; ~ ~ ~ ~ ~ -   14       0

0 ; 34  7  '  ~'! hx  >       0

0~~~~~~          0~~~~~~~4   0

12T                        .

0   ~ ~ ~  ~    4,0   12   02  N~~~~~~~P

'.004  L  ~  0

pq~z

656

2 o  com

0

I.

INDUCTION OF MOUSE TUMOURS BY DYESTUFFS

o    ooC  0  0 0

Co  a  0 C  CO  CO e, CO

0

w

to

1sC             :~~'~ ~ 4  0

.N   ^-;          V aq 0  0 0

*  *i~~~~~~~~~~~- *0 *0 *0 I40

cs     CO  CO  CO ce X X

60 b b b  o

0e
(L)~ ~~~~~C as

*     -   *-  ,*   *

&O O ;      0 0

cO*e         * 44

0 0

4a~~~~4

4~~~~~~~~~~~~~~~*

o   0,'    0l                        eq0 |

N

ON     *
I- >,

0  2-1     i!~~~~-o

10

.;e z
i

657

, 8 -  4 o-

C CO

0 c

0
Ca0

C4. -,-

0 0    d

-4

,      o  U

0

9 e q  co

010o   10

4    V4

colo

-Q
:4:
0 -~

C.) C)

0* *??

;_ coO
0) E

Cr L4
_D 10

OD  - o

0

*-        4

0)        04

k

& ? t?o

I 0 2
02 m

Id

658

GEORGIANA M. BONSER, D. B. CLAYSON AND J. W. JULL

in arachis oil for 52 weeks. Thereafter the mice were allowed to survive as long
as possible. For three reasons, this technique was varied: (a) to compare the
effects of oral and subcutaneous administration in the case of certain chemicals
which might be altered in the alimentary tract or metabolised in the liver; (b)
to avoid inflammation of the subcutaneous tissues by irritant chemioals; and
(c) to reduce labour. The methods of administration, dose, and source of the
chemicals are given in Table III. The chemical formulae of the less well-known
compounds are as follows:

HO          OH

C1H . H,NX   /    \NH, . HCI

(I)

OCH,

41\1/\ NH,. HCI

l 11 1

(UK)

N = N <> 1==>   OH
0/\ JuOH  NaO,S/ (N=-N )

.O,Na

(III)

CH,
N = N  \

/\/     OH    CH3

11
11

(V)

H,N/=\/

CH,

(VII)

(IV)

CH,
N = N
1//     OH

(VI)

HJC           NH,}C1       CH,

> (==S ~~=--//< H
H,C                        OH,

(VIII)

COH

11 CO,H

-1//\/S/

l I 11

H5IC2  l       1   C.Hr5

C1     N     ? \N/

H5c,               C,H5

(IX)

SO,Na

NaO,S//-\ N = N-I==

HO
(X)

SO,Na
CH_H,

CH,/<      N  _X-) / O

(XI)   OH

INDUCTION OF MOUSE TUMOURS BY DYESTUFFS

Post-mortem examination.-At death a thorough examination of each mouse
was made by searching for tumours in all organs. Microscopical examination
of all suspicious lesions and of many apparently normal organs was undertaken.

EXPERIMENTAL RESULTS

The mortality at the beginning of the experiment, which took 3 years to
complete, was very high. Improvement was obtained in some groups by vaccination
against ectromelia.

Subcutaneous sarcomas.-With one exception, these occurred in mice reoeiving
oily subcutaneous injections of the chemicals (Table IV). The 12 subcutaneous
sarcomas induoed with 2-naphthylamine have already been reported and discussed
(Bonser, Clayson, Jull and Pyrah, 1956) as have the three induced by subcutaneous
injection of o-tolylazo-2-naphthol (Bonser, Clayson and Jull, 1954). Of the three
tumours induced in the subcutaneous tissues with benzeneazo-2-anthrol, two
were spindle-cell sarcomas of vaso-formative type (Fig. 1), which resembled
those described by Andervont (1950) as haemangio-endotheliomas following
subcutaneous administration of crystalline o-aminoazotoluene, and one was a
spindle-cell sarooma. They occurred at 34, 46 and 56 weeks in 2 males and 1
female. The three tumours induced with o-tolylazo-2-naphthol were spindle-cell
sarcomas in females, at 46, 53 and 61 weeks respectively, as was the one tumour
occurring at 56 weeks in the flank of a female mouse receiving oral auramine. The
latter was not associated with parasitic infection.

Hepatomas.-A significant incidence of this type of tumour was obtained with
benzidine, 2-naphthylamine, auramine and 0-methyl-2-amino-1-naphthol hydro-
chloride (Table IV). Single tumours were obtained with benzeneazo-2-anthrol
and the injection of o-tolylazo-2-naphthol. Ten hepatomas occurred before and
16 after 70 weeks; in the latter group were 6 induced by 2-naphthylamine. Those
occurring after treatment with benzidine and 2-naphthylamine were large in
size and sometimes multiple. Although no metastases were found, their structure
frequently resembled that of malignant tumours. Those induced by auramine
and 0-methyl-2-amino-1-naphthol were small and benign. It was rare for cholangio-
matous areas to be present in addition to hepatomas.

Lymphomas.-This term is used to cover all the lesions arising in the reticular
system. Using the classification of Dunn (1953), the two common tumours were
lymphosarcoma and reticulum-cell sarcoma. The former varied greatly in its
extent, and might be confined to the liver and spleen, or to the lymph glands,
in an individual mouse. The latter arose usually in the abdominal cavity or in
the liver and kidney. The tumours were composed of spindle cells and it was
necessary to make a distinction between this type of tumour and the spindle-cell
sarcomas arising locally in relation to subcutaneous injeotions. This was not
difficult when the anatomical and microscopical appearances in each mouse were
studied. This statement is in accord with the opinion of Dunn (1953).

Approximately one-third of untreated mice of this type used in this and other
experiments suffer spontaneously from the disease, the females being more
commonly affected than the males. Treatment by the chemicals under review
did not significantly raise this incidence.

Intestinal tumours (Table V).-One year after the beginning of the experiment
an intestinal carcinoma was found in the ileo-caecal region of a mouse injected

45

659

GEORGIANA M. BONSER, D. B. CLAYSON AND J. W. JULL

* "      I 1c = at I I 00

-I _l I O _ C      I I -

. . . . . . . . .

=  - MDt CS aq  t-O

_- aq       r-

= = 00 x la - IC 00

-

F-- 0

. . . . . . .
I C  4a -  aq (

I  Oo   "-  I -- 0

I   I."    I CO
I I Coil I ",

. . . . .

_~ _

104104
I14 r I oo

. . . . .

I I CO I O>

Ci I  m C_  I*  C C  I __
cI eq lq _* C0 _  I-_   1   C

1* - co r to c= _ I -* _ es to

_ M"- _   aq es  I _ Ci I _- CA

- I I  r.lOU:1
---+ I j<l)O J

00 000   0

coeC de00C   ed

* * * .0

I oo I I I'"1-

101I00 1001

* . . . . . . . . . .

I  40 C>

C O IC

1 00

I( 00
I1 00

IU     I   I I  I I o   -I I--  I  o

IS I C I I 01 JiC~    I ~0
I Co   I m  t-   I  I G; .* _   I l  = cq =c

I m   I --I.,* -4 -4-*m   I eq  m   I m   1

I  Ied  I  o

I ed1- 1 1 - ?;*

0* 0. 00. 000 ... *

* d  C* e . C.  * .

o   o    o

e 0 c0Xc   c  e

I"-I Iq _-
I  I"  I    I

.    .   .   . *.

00           0

C5 Cd      Ca

*   *     *   I

C r .F

*-   0..

"~~~~~- ~ ~ ~ ~ -

r e d

.

6        1     1

4         .     .

1-4
0
?o

4--?        0
P-1      , r-4

ce         10

4-'-)

5           0

4          ce
6        1 :?,

0
.,.q

E Q 2 N

't 'O -

.-q m    4)

r
C,l 0    C) 1j)

I" ? N

4

C) r-4 0
i      cd  4-i

um

0 r

44 -~~

P-

660

o o
rd |~

o rx
ad ^i

fX

-o
0

m
1!4

8
r.

bo
0

;>.4
q?
4)

Q

. 1-4

I

cIt

14)

t3

co

I
Eq

Mj

0

o 0 C4E

~ o

II rt C.

- 3 0

Fl)

100

'4

44      0

S o d .

C5

661

INDUCTION OF MOUSE TUMOURS BY DYESTUFFS

*S 4           ao to xo>oo  oo

o>

m~~~~o -x

*      . * *

~~~~C        .5 (Z  C)  C>

*~~~~~            o

X *~~~~~a      m  c, qC#

0                     C1

.>  e  S    -4     c V

..4 x    I  I4 Ia xo o

eql   I   I  IIoI

**.*.

..J

04

o

*    c)

OD

eQ

4*-4

o s sq Q  0s

O C) r   C> ut O > 0s e

O O  _ G

1-4 ~ ~ ~

I- Iv  I  IN _I

0

I . 1, 1  o   1

4D 4

0o  l  lo o

"I l o

0  :  c

C._

0 d
o

9D.

'P 4

P o
OD

..
0.

cs

GEORGIANA M. BONSER, D. B. CLAYSON AND J. W. JULL

with o-tolylazo-2-naphthol. Thereafter, the intestinal tract was searched more
thoroughly for possible tumours and all suspicious areas were examined micro-
scopically. In addition to cutting sections of the ileo-caecal region (Table V),
259 pieces of small intestine and 93 of large intestine were cut and examined.

It was decided to accept as without demonstrable action on the intestinal
tract those compounds where one intestinal tumour or no tumours were present
in the group. These are: arachis oil, benzidine, dihydroxybenzidine, 1-naph-
thylamine, auramine, magenta, carmoisine, benzeneazo-2-anthrol and sunset
yellow. With these chemicals the incidence of polyps (only one of which, in an
auramine mouse, was malignant) was 4 out of 31 males and 2 out of 49 females
(Table V). The remaining compounds, i.e. 2-naphthylamine, 0-methyl-2-amino-1-
naphthol hydrochloride, rhodamine B, xylylazo-2-naphthol, tolylazo-2-naphthol
and ponceau 2R, each produced more than one intestinal tumour in the group.
In total, there were 23 intestinal tumours in 49 males and 10 in 40 females (Table
VI). Of these, five tumours were intestinal carcinomas.

Morbid anatomy and histology.-The site of election was the ileo-caecal junction
(35 out of 39 tumours). Where the precise origin was detectable, it was usually
from the wall of the caecum, but some tumours seemed to start exactly at the
junction and others were multifocal, arising both at the junction and elsewhere.
One benign polyp arose in the ileum at a little distance from the junction, and
another in the colon near to the junction with the caecum. The site of two
carcinomas was in the colon about 1- inches from the anus. The tumours could be
detected either by observation of a thickening of the region or by the presence of
small shining cysts on the serosal surface. Some of the small tumours were not
deteoted with the naked eye.

Structure of the polyps.-These were usually sessile adenomas, raised from the
mucous surface and composed of regular intestinal glands, lined by high mucus-
secreting epithelium, in which the nuclei are markedly hyperchromatic (Fig.
2 and 3). Dipping of the glands through the muscularis mucosae and the muscular
wall of the intestine into the mesentery was a frequent feature causing the cysts
seen externally with the naked eye (Fig. 4). Other cysts were formed by oblique
cutting of pockets of the intestinal lumen caused by the projecting polyp and these
sometimes contained faeces or portions of tapeworm. Sometimes there was a
granulomatous reaction in the mesentery, thought, to be due to the bursting of
one or more of the cysts. Similar tumours were described by Miller and Pybus
(1956).

Structure of the carcinomas.-All but one of the 6 tumours of this type could
be seen to arise in pre-existing polyps. In 2, there was no muscle invasion (Fig.
5); in 2 there was complete penetration of the musoular coat by mucus-forming
irrregular cystic glandular spaoes; in 2 there was anaplastic invasive growth
penetrating into the mesocaecum and associated with serosal lymphatic permeation
and metastasis to regional lymph nodes (Fig. 6). Adjacent to both these tumours
were benign cysts of the type described above as forming the deep part of benign
papillomas of the caecal region. The finding of these cysts was regarded as evidence
of the previous presence of a benign tumour.

Pyloric polyposis.-This is a proliferative condition associated with over-
growth of pyloric glands, which penetrate the muscularis mucosae and the muscular
coat, but do not invade lymphatic vessels nor metastasise. This condition is
spontaneous in the I and other strains (Stewart and Andervont, 1938) and has

662

INDUCTION OF MOUSE TUMOURS BY DYESTUFFS  663

00  0  -0 0  0
C)

0

0    ---  t0  0 o -  -

00

0

0

0  0

fi Q~~~~~~~~~~~~~~~~~~~~4
I ~~~~  0 0-0 0 0~~~Z0 ~ 0
" m z

~~~~) ~~~~~~~~~-  -~ ~ ~ ~~-p

21     P4ow{

0

0                  00 0  0

P4.5

| V ~ , i n  ;
E i  v   c ?  -   0   C   0  1

0~~~~~~0

Co      o

0~~~~

N ~~    -m

.C - 20   0          0 '0 . .

P  .-.  s            - l   -   _i

04

o        - cEI

V ~ ~~~ . fr *11 '  C ,

GEORGIANA M. BONSER, D. B. CLAYSON AND J. W. JULL

recently been described by Miller and Pybus (1956) in older mice of crosses
between the NBT and CBA strains. The lesion ocourred in control and treated
mice of the present experiment, and was more oommon in males than in females.
It is usually non-malignant, though Miller and Pybus described one tumour
which they thought was an adenooarcinoma.

Other tumours.-A squamous carcinoma of the orbit occurred at 63 weeks in
a male mouse injected with an oily suspension of benzeneazo-2-anthrol. A
squamous papilloma of the lower end of the oesophagus and a squamous carci-
noma of the cardiac end of the forestomach also occurred in 2 females at 58 and
56 weeks respectively in the group treated wth this chemioal. Ocoasional mammary
carcinomas and granulosa-cell ovarian tumours occurred in control and other
groups. One spindle-oell sarooma of the uterus ocourred at 70 weeks in the
xylylazo-2-naphthol group and one osteogenic sarcoma of the jaw at 74 weeks in
a female treated with sunset yellow.

DISCUSSION

Single early benign intestinal polyps were found in groups of mice treated
with arachis oil, 3: 3'-dihydroxybenzidine, 1-naphthylamine (free from 2-naph-
thylamine), and carmoisine. One small hepatoma was seen at 101 weeks in a female
treated with magenta and an osteogenic sarcoma of the jaw occurred in a mouse
treated with sunset yellow. As all these tumours might well be casual occurrences
these six compounds are regarded as being without carcinogenic activity in this
experiment. Case and Pearson (1954) stated that the manufacture of magenta
had an " occupational hazard of causing tumour of the urinary bladder attached
to it." No carcinogenic properties have been demonstrated in magenta in the
present experiment, where a high dose was used but only a few mice survived
to a late period.

Baker (1950) claimed that 3: 3'-dihydroxybenzidine, recovered from the urine
of industrial workers, was carcinogenic to the mouse when given subcutaneously
in oily solution, producing benign and malignant tumours in the bladder and the
liver. As the author stated that the chemical was not completely purified, as
benign and possibly malignant hepatomas were also observed in untreated control
animals and as the illustrations of the tumours are quite inconclusive, the value
of the observations is doubtful.

Case, Hosker, McDonald and Pearson (1954) brought evidence to show that
1-naphthylamine was a cause of bladder tumours in industry. They thought it
possible that the 2-naphthylamine content, always present in crude samples,
was not the sole causative agent of the industrial tumours. The present experi-
ment brings no further evidence that the purified compound is carcinogenic
either to the bladder or to any other organ.

The following compounds were regarded as showing carcinogenic activity:
benzidine, 2-naphthylamine, auramine, o-tolylazo-2-naphthol and benzeneazo
2-anthrol.

Benzidine was shown by Spitz, Maguigan and Dobriner (1950) to induce
hepatomas; intestinal carcinomas and acoustic duct carcinomas in the rat.
Baker (1953) was also able to induce these types of tumour in Slonaker rats, and
he claimed 2 squamous carcinomas of the forestomach and one papilloma and 2
squamous carcinomas of the urinary bladder. Tumour induction in the dog
requires a long latent period and yields only a low incidence of tumours. Spitz

664

INDUCTION OF MOUSE TUMOURS BY DYESTUFFS

(personal communication) found that the 3 longest survivors of 7 dogs given
large doses of benzidine by mouth developed papillomas and carcinomas of the
bladder after 7, 8 and 10 years respectively. Of 4 dogs treated similarly in Leeds,
two survived for 81 and 9 years and had no bladder tumours at death (unpublished
observation). An invasive bladder carcinoma was induced after 21 years in one
rabbit in Leeds (unpublished observation), among 7 treated by oral administra-
tion of benzidine. Baker (1950) described 5 hepatomas-4 benign and 1 malignant
-in 9 mice of Gidham stock which had received subcutaneous injections of an
oily suspension of 300 mg. of benzidine base 3 times per week for 45 weeks. Five
of 17 untreated mice developed 3 benign and 2 doubtfully malignant hepatomas
in the same time period. The dose reported by Baker was found in the present
experiment to be greatly in excess of the maximum which could be tolerated by
our mice. Our tumours were confined to the liver. They occurred in both sexes
between 50 and 79 weeks of treatement.

The tumours induced with 2-naphthylamine have already been discussed
(Bonser et al., 1956) and reasons given for regarding the subcutaneous sarcomas
as due to the development of carcinogenic properties in oily solutions on standing.
Hepatomas occurred even when the water-soluble hydrochlorides were injected
and this was regarded as being due to the action of 2-naphthylamine or its meta-
bolites on the liver. All but one occurred after 70 or more weeks of treatment,
i.e. late in the experiment. In addition, one benign polyp and one carcinoma
arose at the ileo-oaecal junction.

The claim for carcinogenic properties in auramine lies in the 7 hepatomas
(4 in males and 3 in females) occurring in 19 mice. The one carcinoma of the
colon in a male mouse treated for 67 weeks is regarded as probably fortuitous
and the origin of the subcutaneous sarcoma following oral administration is
unexplained, though it might have been caused by the chemioal.

o-Tolylazo-2-naphthol was administered by injection and feeding. One small
intestinal polyp and 2 carcinomas occurred after injection (Bonser, Clayson and
Jull, 1954) and 9 polyps and one oarcinoma after feeding. In the latter group,
only the males were affected and all the polyps but one were at the ileo-caecal
junotion and were of the characteristio cystic type. In the benzeneazo-2-anthrol
group, were 3 subcutaneous sarcomas, 2 of vaso-formative type, usually classified
by other authors as haemangio-endotheliomas (Foulds, 1930; White and Stewart,
1942; Andervont, 1950). White and Stewart suggested that their tumours
occurred along the line of drainage of oral methylcholanthrene from the intestinal
tract. The present tumours could either be regarded as occurring at the site of
injection of the chemical or in tissues likely to be receiving lymph from the injected
area. These were the only tumors of this type in the whole experiment and there-
fore they are regarded as being specifically related to this chemical. One squamous
papilloma and one squamous carcinoma of the forestomach, and one squamous
carcinoma of the orbit were also observed in the group treated with benzeneazo-
2-anthrol. The significance of the last-named tumour is doubtful.

0-Methyl/-2-amino-1-naphthol hydrochloride, xzylylazc-2-naphthol, ponceau
2R and rhodamine B are chemicals of which the carcinogenic activity is in doubt.
When the mioe were treated with 0-methyl-2-amino-1-naphthol hydrochloride by
subcutaneous injection, 2 hepatomas were found in male mioe but the outstanding
feature was the 8 benign polyps, all situated at the ileo-caecal junction and all
diagnosed on naked-eye examination of the exterior by the presence of subserous

665

GEORGIANA M. BONSER, D. B. CLAYSON AND J. W. JULL

and intra-muscular cysts. These occurred between 55 and 78 weeks, i.e. well
within the age when carcinomas had developed in other groups. Therefore,
although the high incidenoe of benign tumours suggests that the compound is
not without tumour-inducing properties, the absenoe of malignant change leaves
its carcinogenic nature in doubt. Xylylazo-2-naphthol was administered by
injection and feeding. No intestinal tumours occurred in 5 injeoted survivors
but there was one hepatoma; 4 benign intestinal polyps were induced when the
dye was fed. These were found at the ileo-caecal junction and 3 of them showed
the characteristic cysts. Four benign caecal polyps were induced in males and
females with ponoeau 2R.

In regard to the intestinal tumours as a whole, there was a marked predi-
lection for the male sex (27 out of 80 males and 12 out of 89 females). There was
histological evidence that carcinomas arose in polyps, but none of the polyps in
mioe treated with 0-methyl-2-amino- l-naphthol hydrochloride, xylylazo-2-naph-
thol or ponoeau 2R became malignant within the period of the experiment. The
preponderance of tumours at the ileo-caecal junction suggests that stasis of the
intestinal contents is a factor concerned in carcinogenesis.

SUMMARY

An experiment is described in which stock albino mice, bought from a dealer,
were used to test the carcinogenic properties of 14 chemicals and the vehicle
of administration.

The chemicals were either: (1) dyes (auramine and magenta), the manufacture
of which constitutes an industrial hazard; (2) dyes (carmoisine, rhodamine B,
sunset yellow, ponceau 2R, xylylazo-2-naphthol and tolylazo-2-naphthol)
formerly or at present in use as food colorants; (3) dyestuffs intermediates
(benzidine, 1-naphthylamine and 2-naphthylamine); or (4) ortho hydroxyamines
(3: 3'-dihydroxybenzidine  and  0-methyl-2-amino-1-naphthol hydrochloride)
related to known metabolites of aromatic amines.

The chemicals were administered either by subcutaneous injection of oily
solutions or suspensions, or orally in the diet, in the drinking water or by stomach
tube.

The carcinogenic activity was assessed by the occurrence of subcutaneous
sarcomas, hepatomas, and intestinal polyps and carcinomas. Lymphomas and
pyloric polyposis occurred spontaneously and their incidence was not enhanced
by the treatment.

Arachis oil, 3: 3'-dihydroxybenzidine, 1-naphthylamine (free from 2-napth-
thylamine) and carmoisine induced no tumours. The one hepatoma obtained with
magenta, and the one osteogenic sarcoma of the jaw with sunset yellow were
regarded as fortuitous occurrences, not related to the treatment.

Benzidine, 2-naphthylamine and auramine were regarded as carcinogenic on
the grounds of the occurrence of hepatomas. Two caecal polyps, one benign
and one malignant, in mice treated with 2-naphthylamine and one carcinoma of
the colon in an auramine-treated mouse were regarded as probably induced by
the chemical. The cause of the one subcutaneous sarcoma after oral administration
of auramine is unexplained. o-Tolylazo-2-naphthol caused 13 intestinal tumours,
10 benign and 3 malignant, in 26 mice. The degree of malignancy of the tumours
in this group was greater than with any other chemical, lymph-node metastases

666

INDUCTION OF MOUSE TUMOURS BY DYESTUFFS                    667

being present in 2 cases. There was a marked predilection for the male sex.
Intestinal tumours followed both feeding and subcutaneous injection, the latter
method being the cause of 3 subcutaneous sarcomas. Benzeneazo-2-anthrol
injected subcutaneously in oil caused 3 subcutaneous sarcomas, 2 of which were
of unusual vasoformative type, one squamous papilloma and one squamous
carcinoma of the forestomach, and a squamous carcinoma of the orbit, the latter
probably fortuitous.

The carcinogenicity of the remaining chemicals, i.e. xylylazo-2-naphthol,
0-methyl-2-amino-1-naphthol, ponceau 2R and rhodamine B remains in doubt,
the first three because none of the intestinal polyps had progressed further than
the benign stage and the last because, although one tumour was malignant,
only 2 tumours were found in 8 mice.

We wish to thank Messrs. L. J. Pointing & Son, Ltd., Hexham, for the gift
of the food-colorants and Professor Bergel, Chester Beatty Research Institute,
for the gift of pure 2-naphthylamine.

REFERENCES

ANDERVONT, H. B.-(1950) J. nat. Cancer Inst., 10, 927.

BADGER, G. M., COOK, J. W., HEWETT, C. L., KENNAWAY, E. L., KENNAWAY, N. M.,

MARTIN, R. H. AND RoBINSON, A. M.-(1940) Proc. Roy. Soc. B., 129, 439.
BAKER, K.-(1950) Acta Un. int. Cancr., 7, 46.
BAKER, R. K.-(1953) Cancer Res., 13, 137.

BONSER, GEORGIANA, M.-(1943) J. Path. Bact., 55, 1.

Idem CLAYSON, D. B. AND JULL, J. W.-(1954) Nature, 174, 879.
Iidem AND PYRAH, L. N.-(1956) Brit. J. Cancer, 10, 533.

CASE, R. A. M., HOSKER, MARJORIE, E., MCDONALD, DREVER B. AND PEARSON, JOAN

T.-(1954) Brit. J. industr. Med., 11, 75.

Idem AND PEARSON, JOAN T.-(1954) Ibid., 11, 213.

DUNN, THELMA, B.-(1953) J. nat. Cancer Inst., 14, 1281.

FOULDS, L.-(1930) Sci. Rep. Cancer Res. Fd., Lond., 9, 89.-(1947) Brit. J. Cancer, 1,

172.

LORENZ, E. AND STEWART, H. L.-(1940) J. nat. Cancer Inst., 1, 17.-(1941) Cancer

Res., 1, 743.

MILLER, ELIZABETH W. AND PYBUS, F. C.-(1956) Brit. J. Cancer, 10, 89.

MURRAY, J. A.-(] 905) Sci. Rep. Cancer Res. Fd., Lond., 2, 47.-(1908) Ibid., 3, 71.
SLYE, MAUD.--(1924) J. Cancer Res., 8, 240.

Ide'm, HOLMES, HARRIET F. AND WELLS, H. G.---(1917) Ibid., 2, 401.

SPITZ, SOPHIE, MAGUIGAN, W. H. AND DOBRINER, K.-(1950) Cancer, 3, 789.
STEWART, H. L. AND ANDERVONT, H. B.-(1938) Arch. Path., 26, 1009.
STRONG. L. C.-(] 941) Cancer Res., 1, 743.

WHITE, J. AND STEWART, H. L.-(1942) J. nat. Cancer Inst., 3, 331.

				


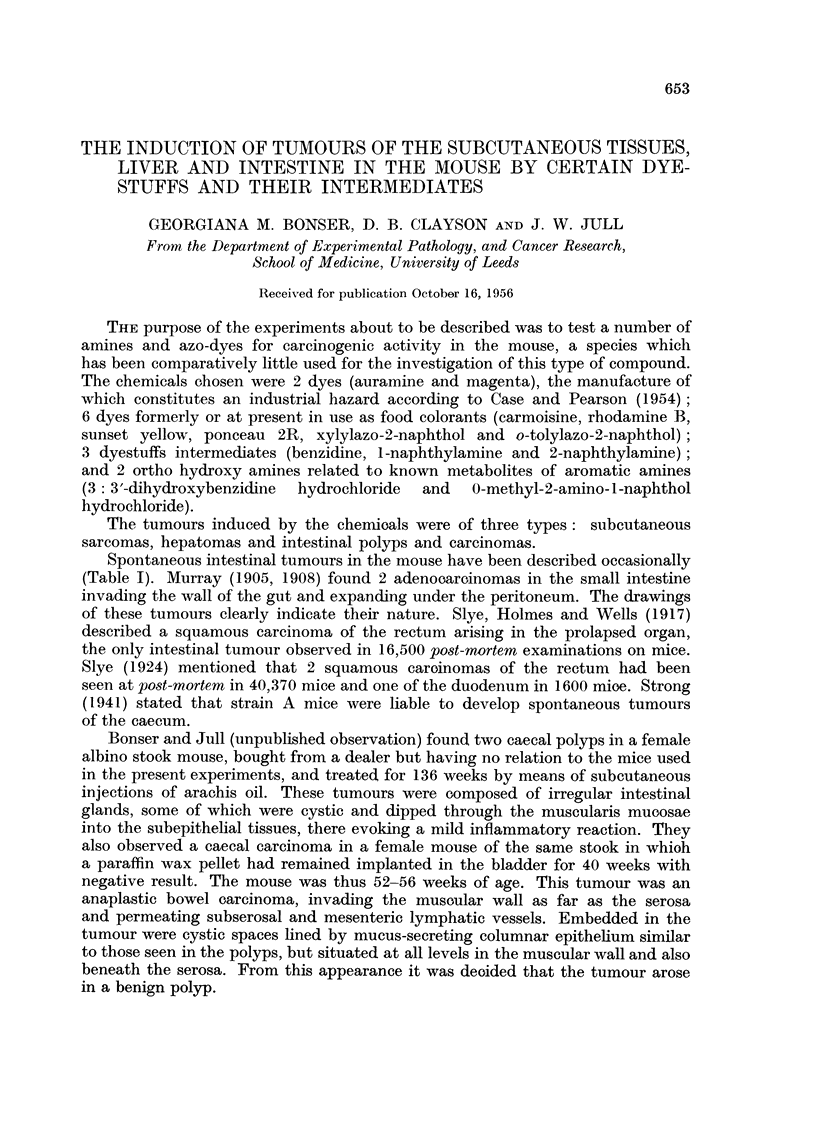

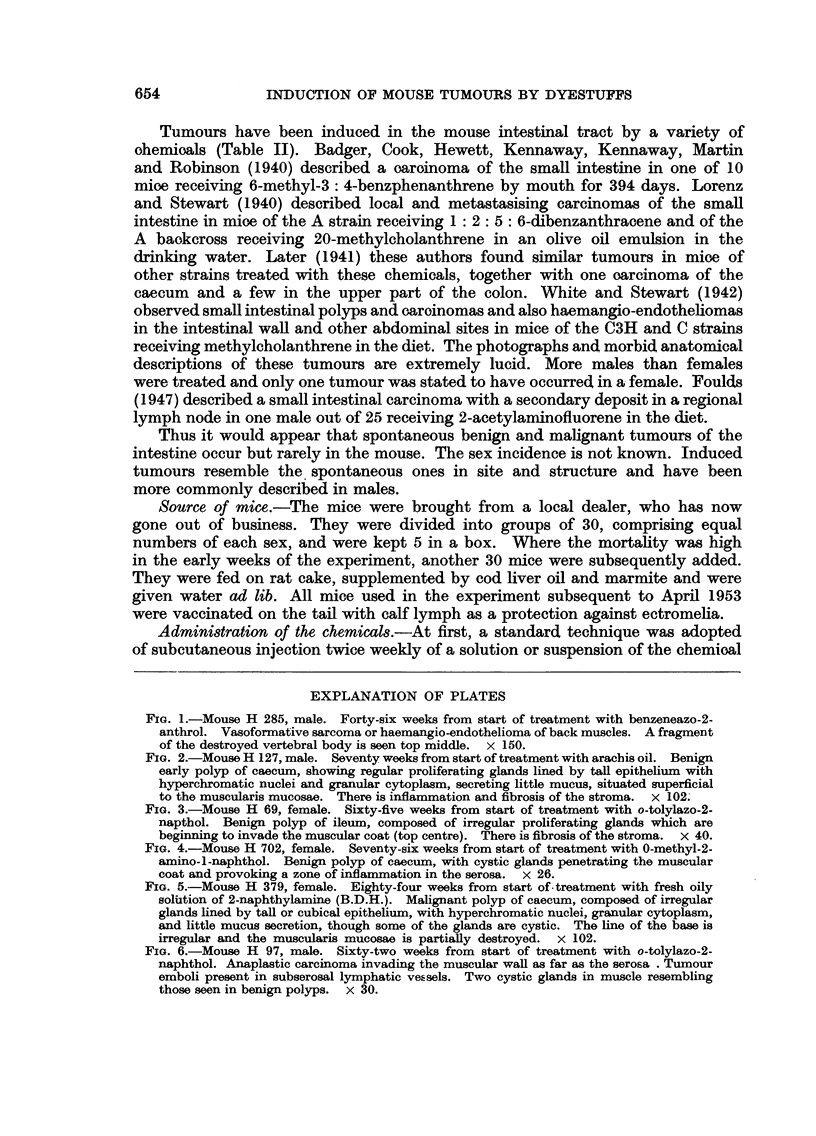

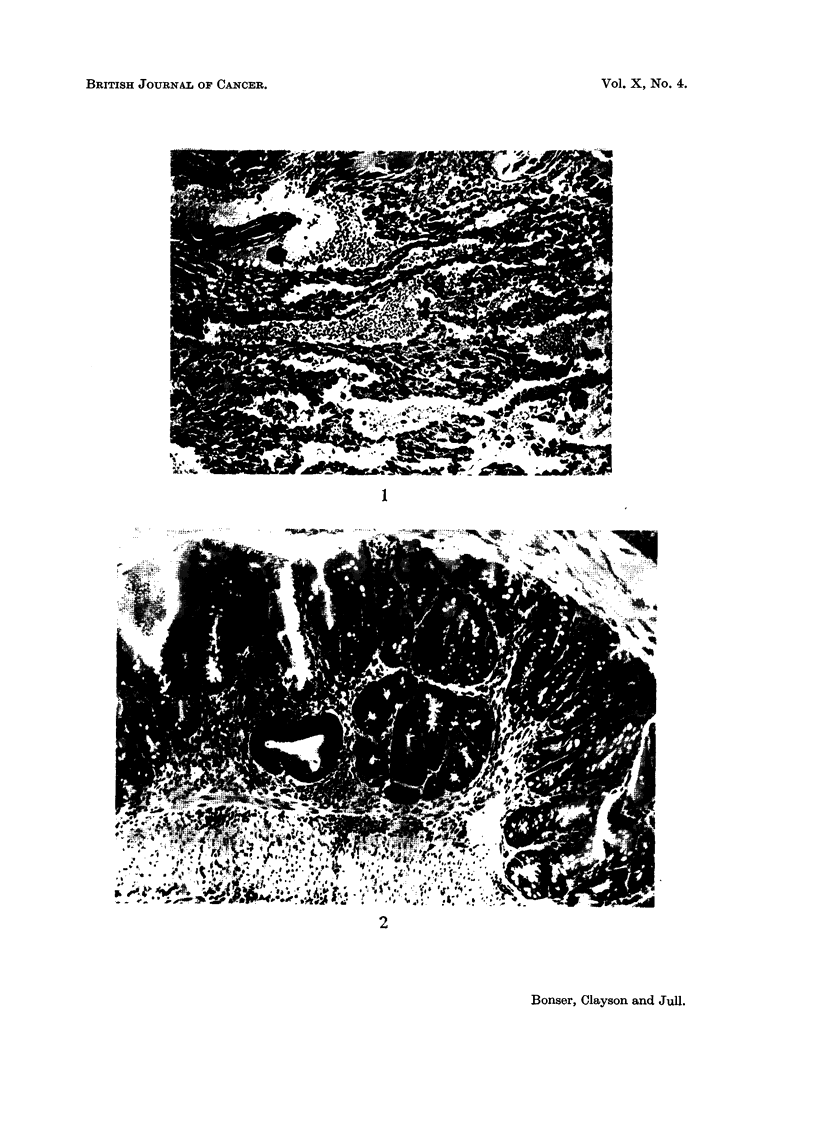

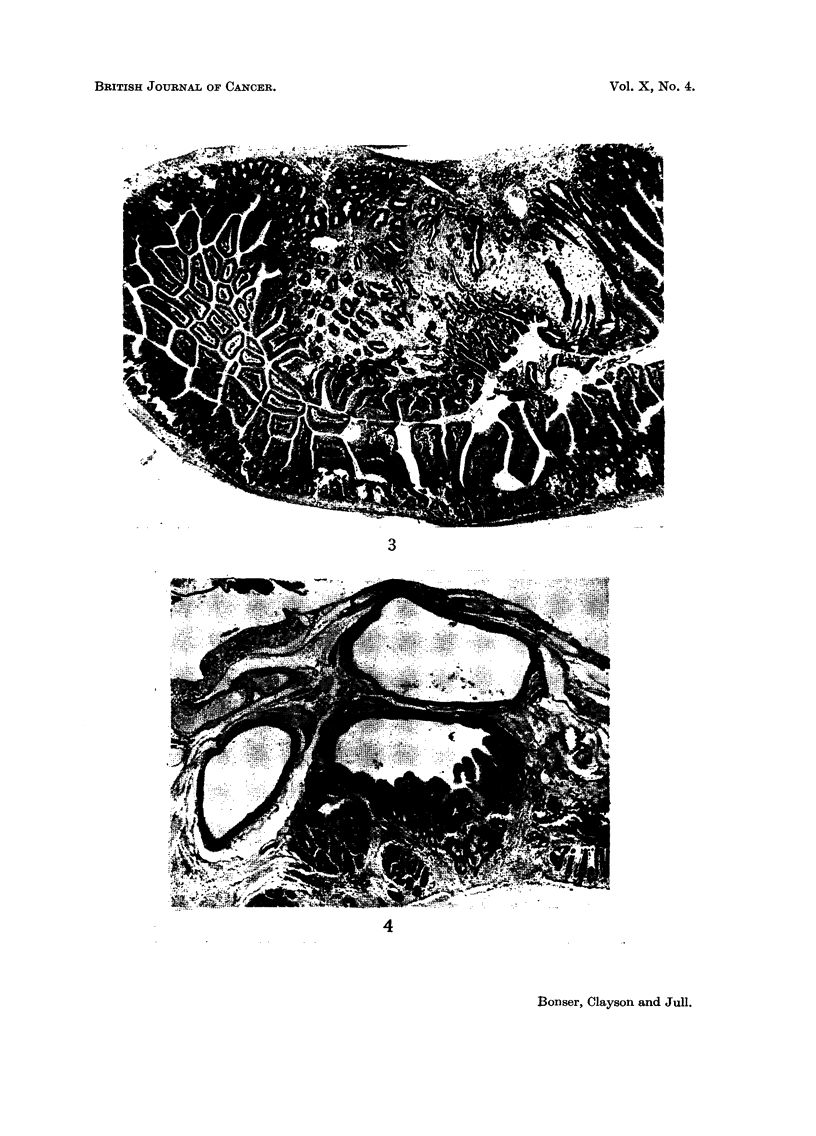

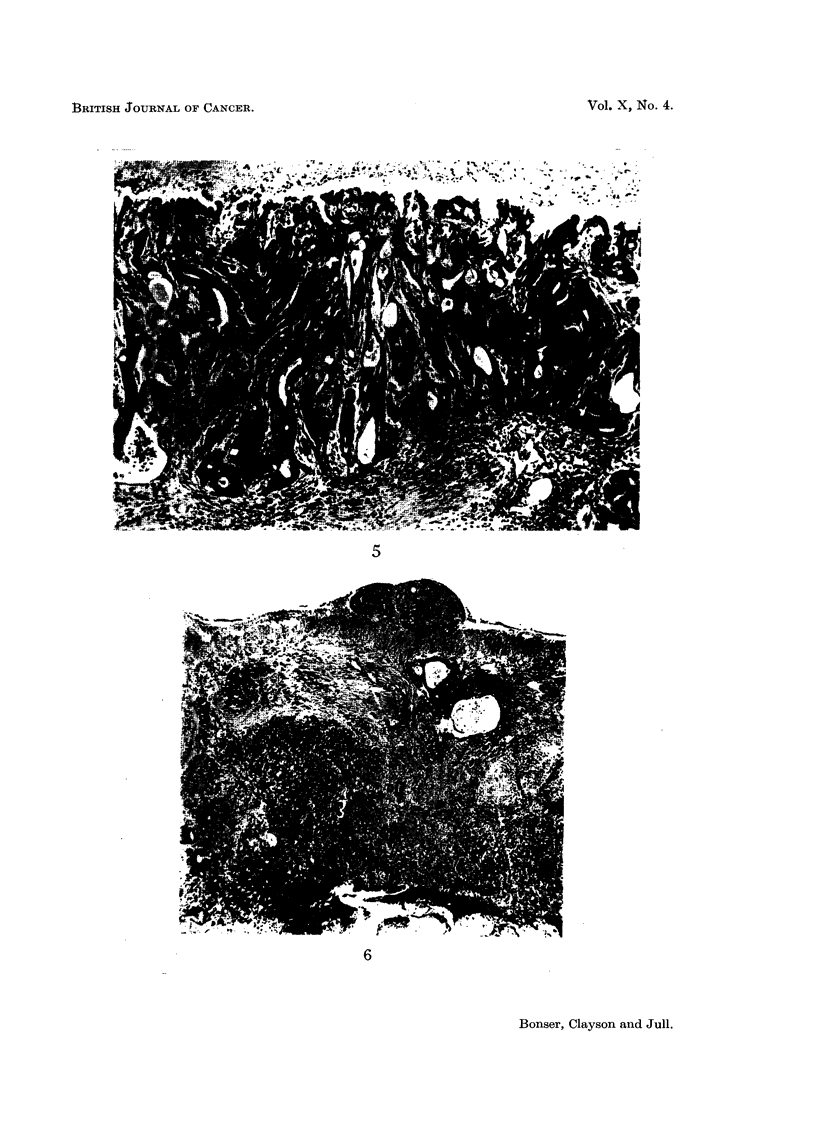

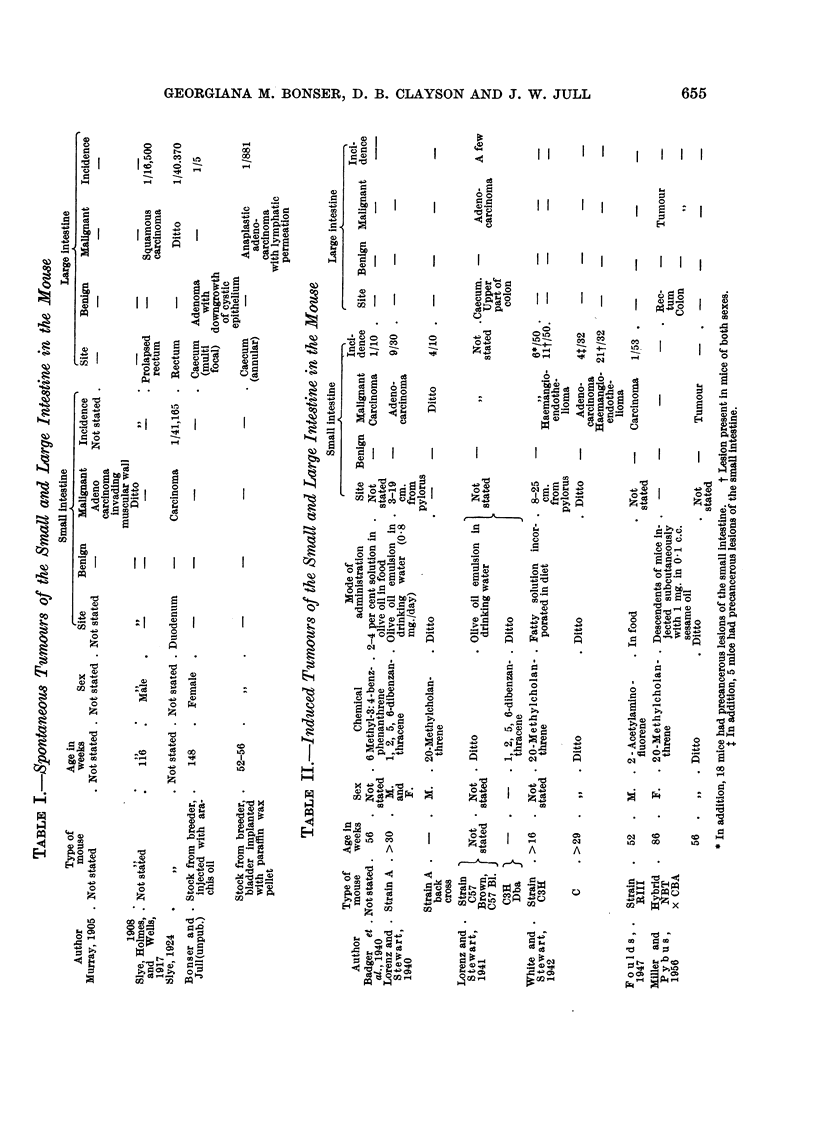

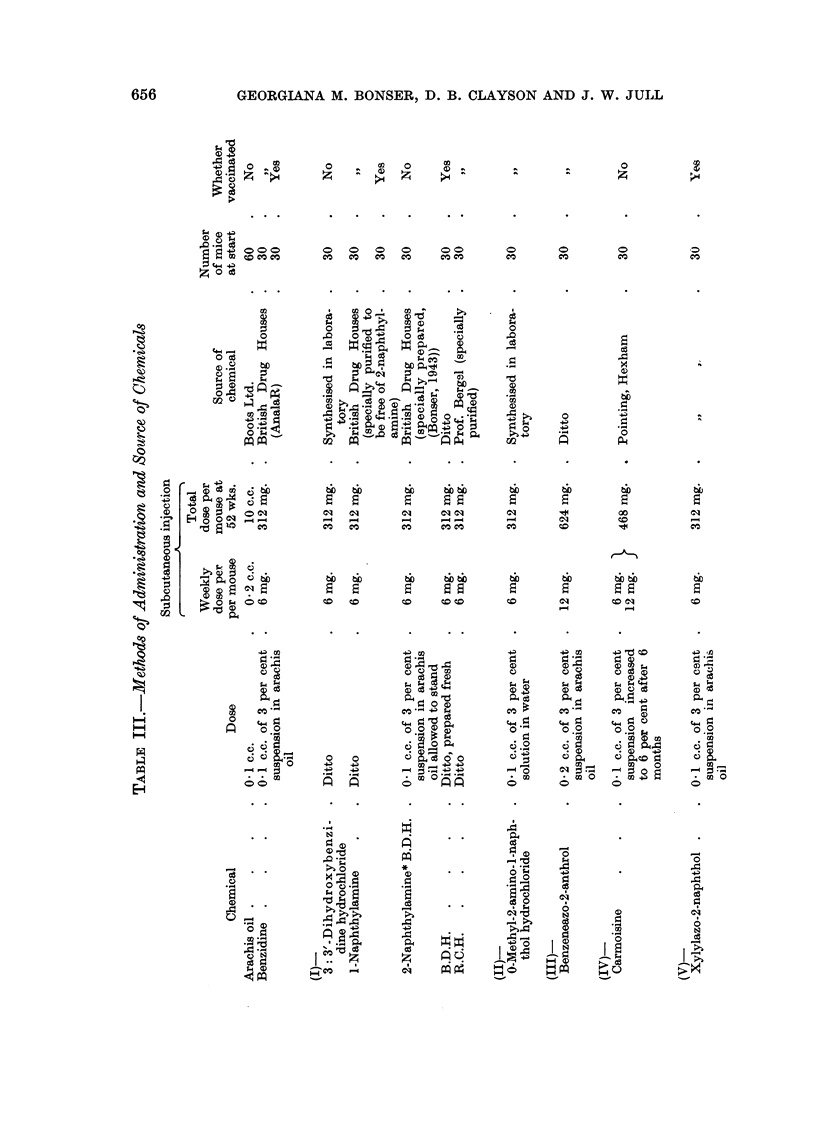

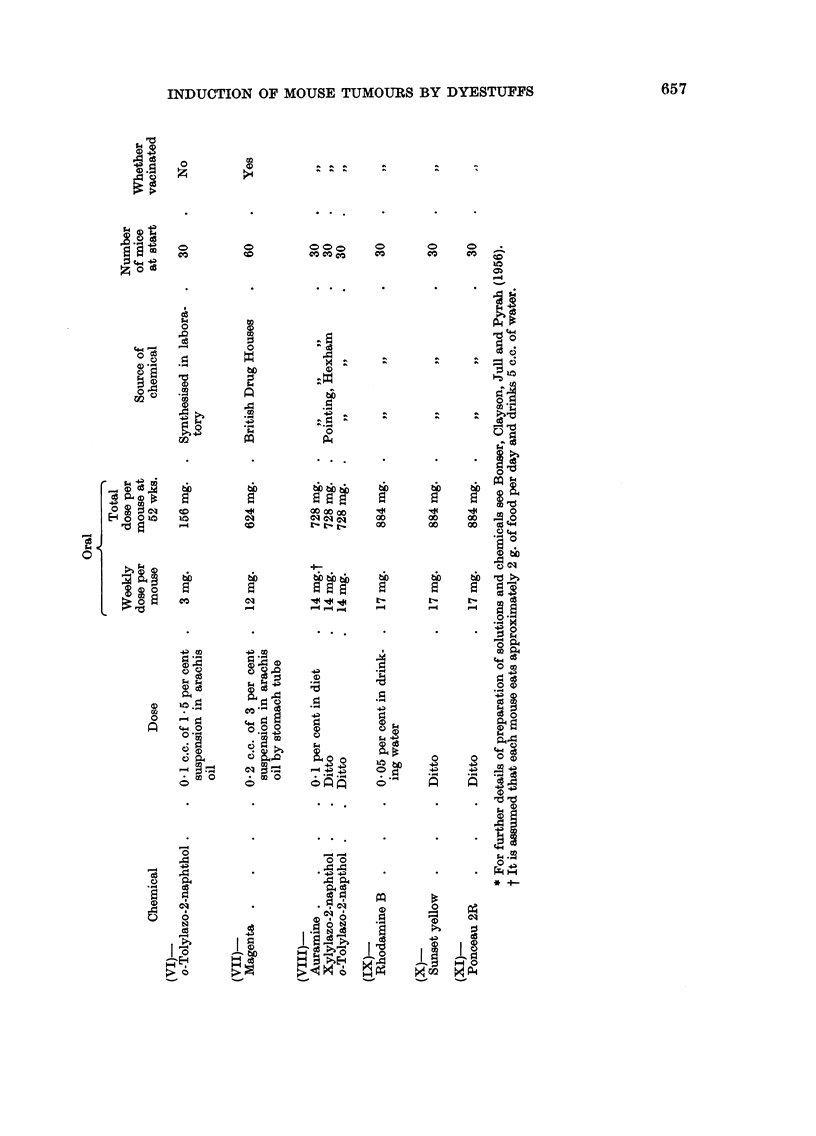

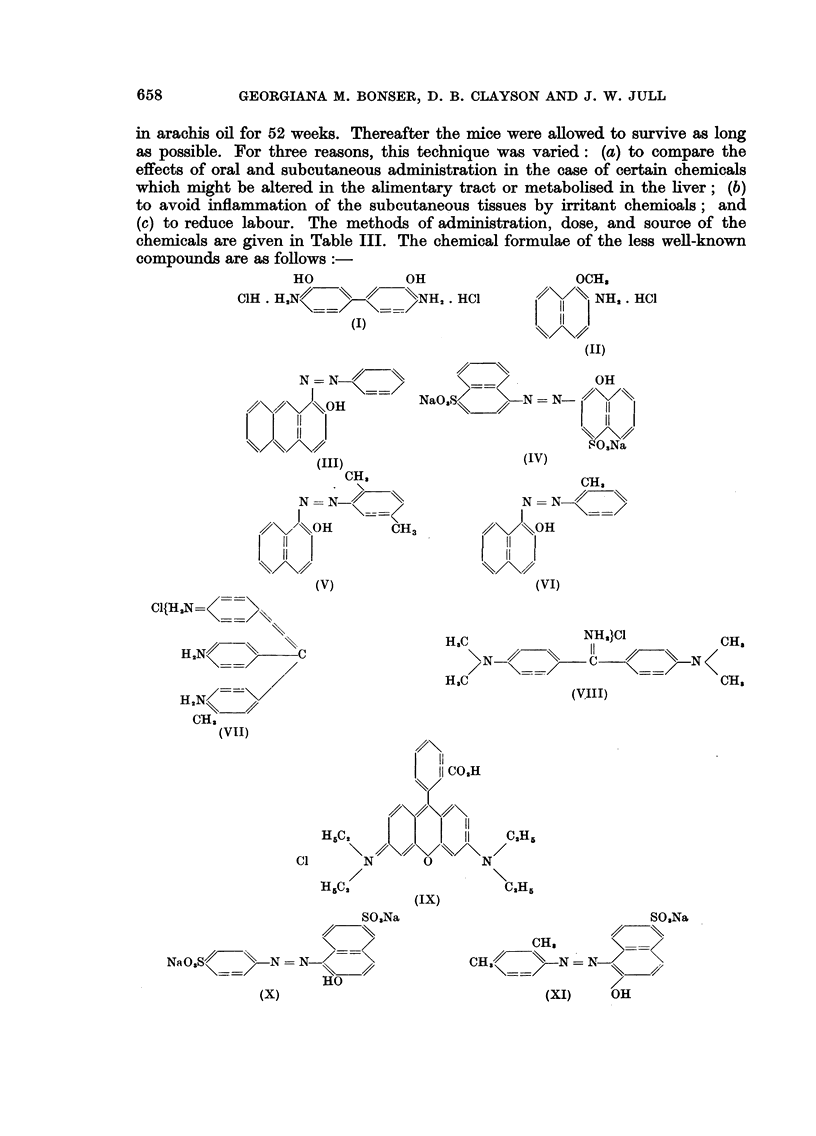

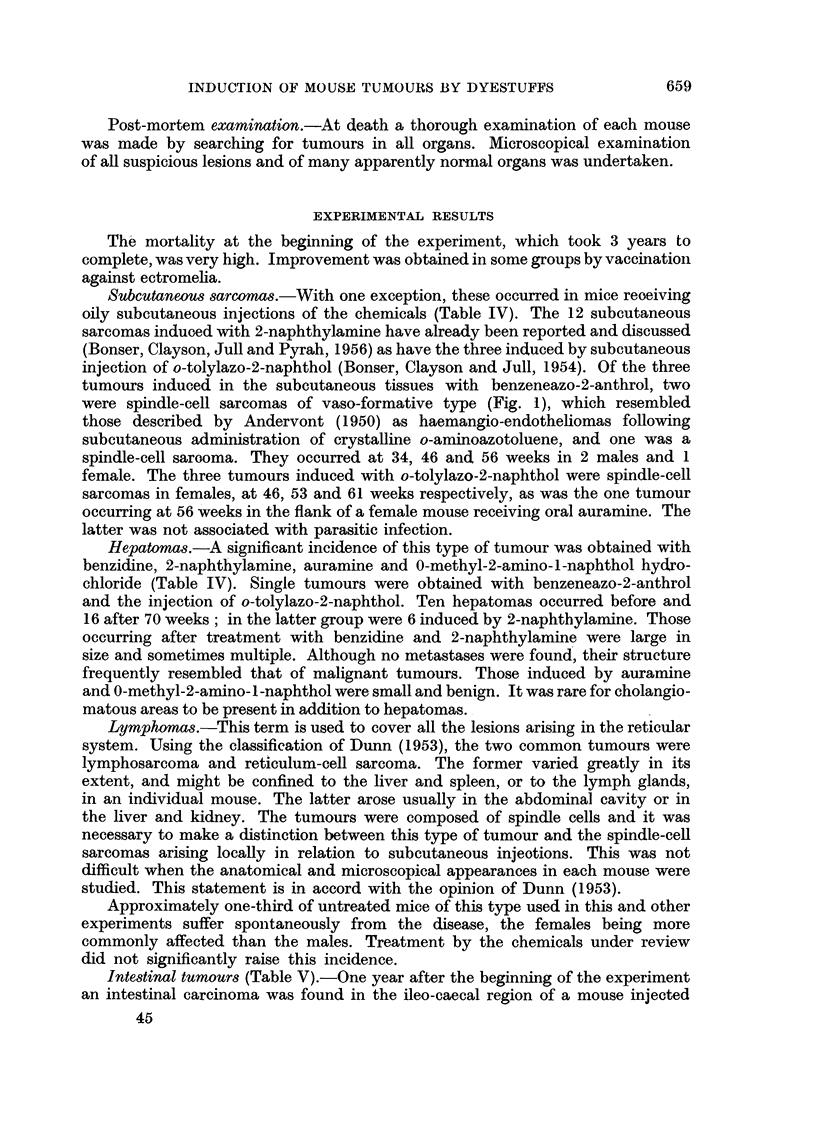

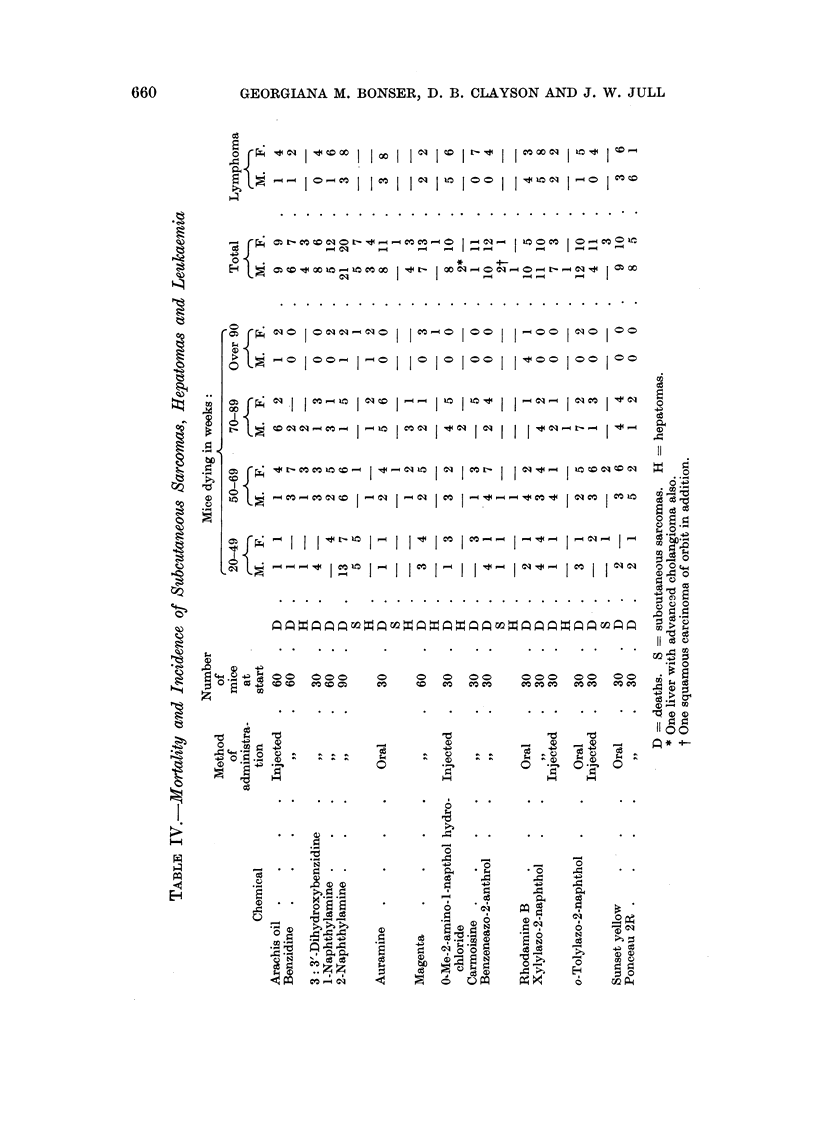

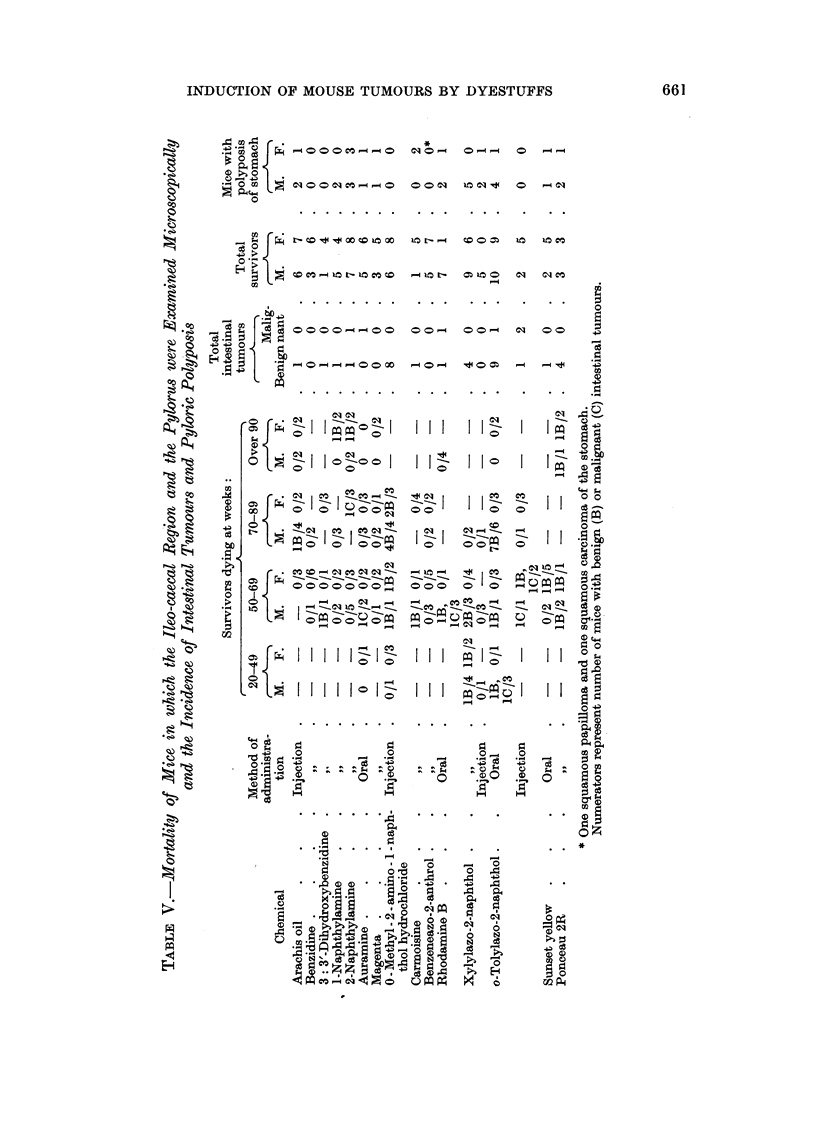

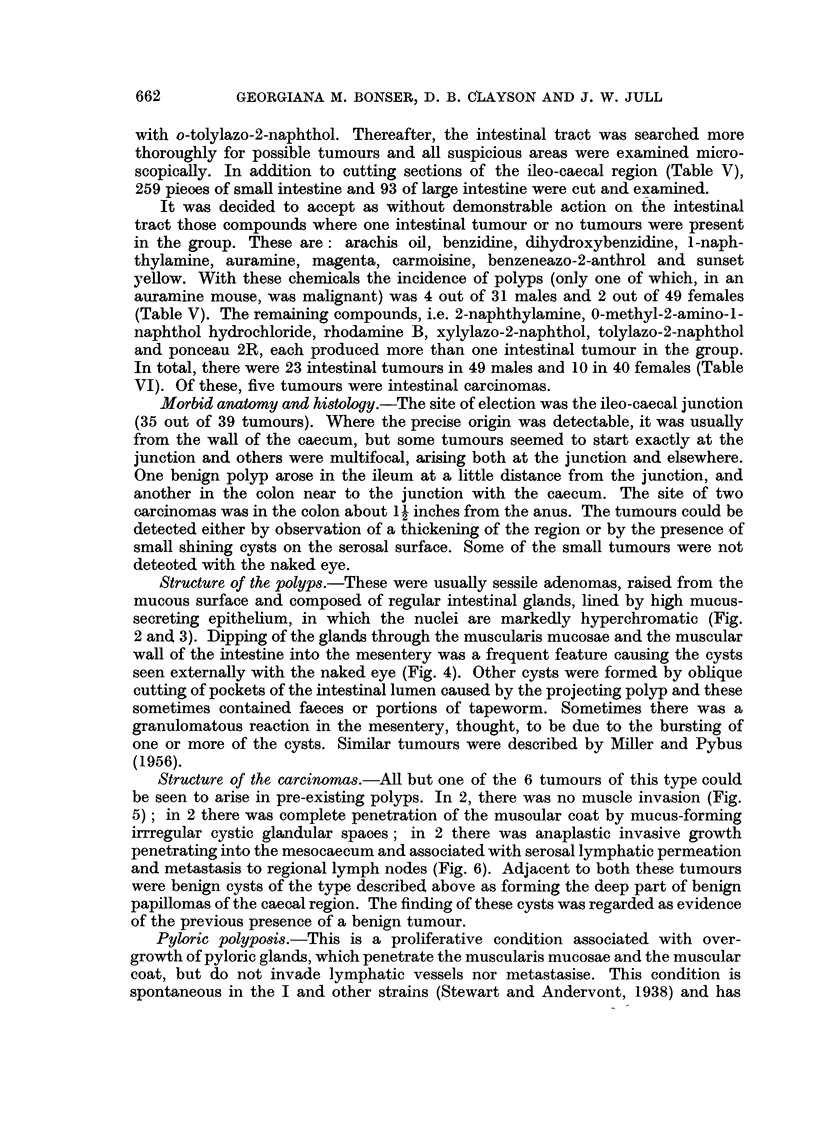

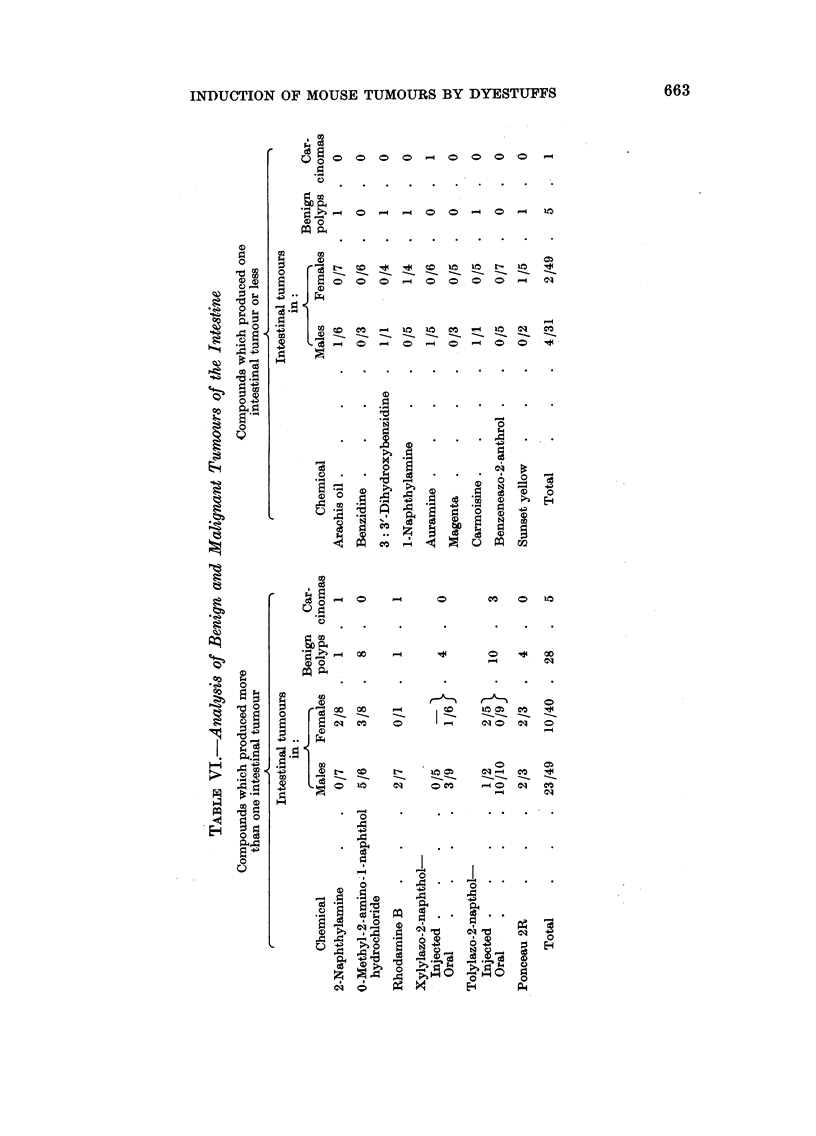

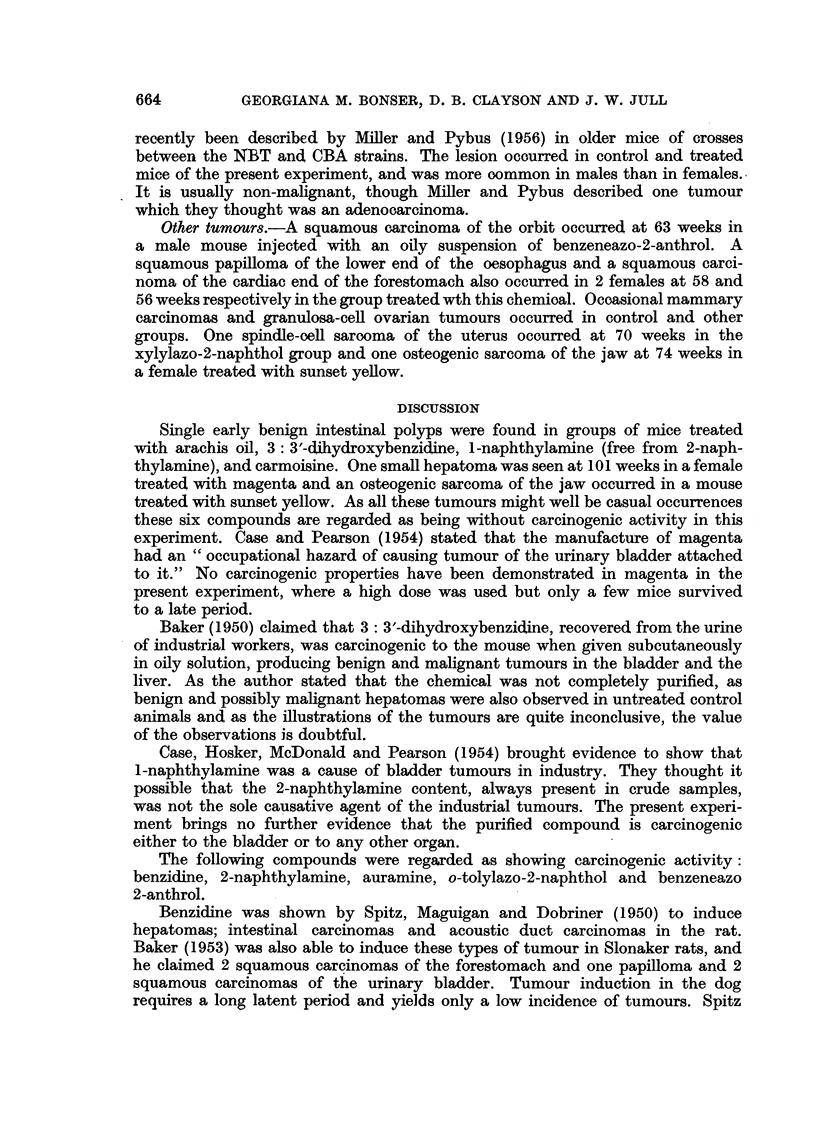

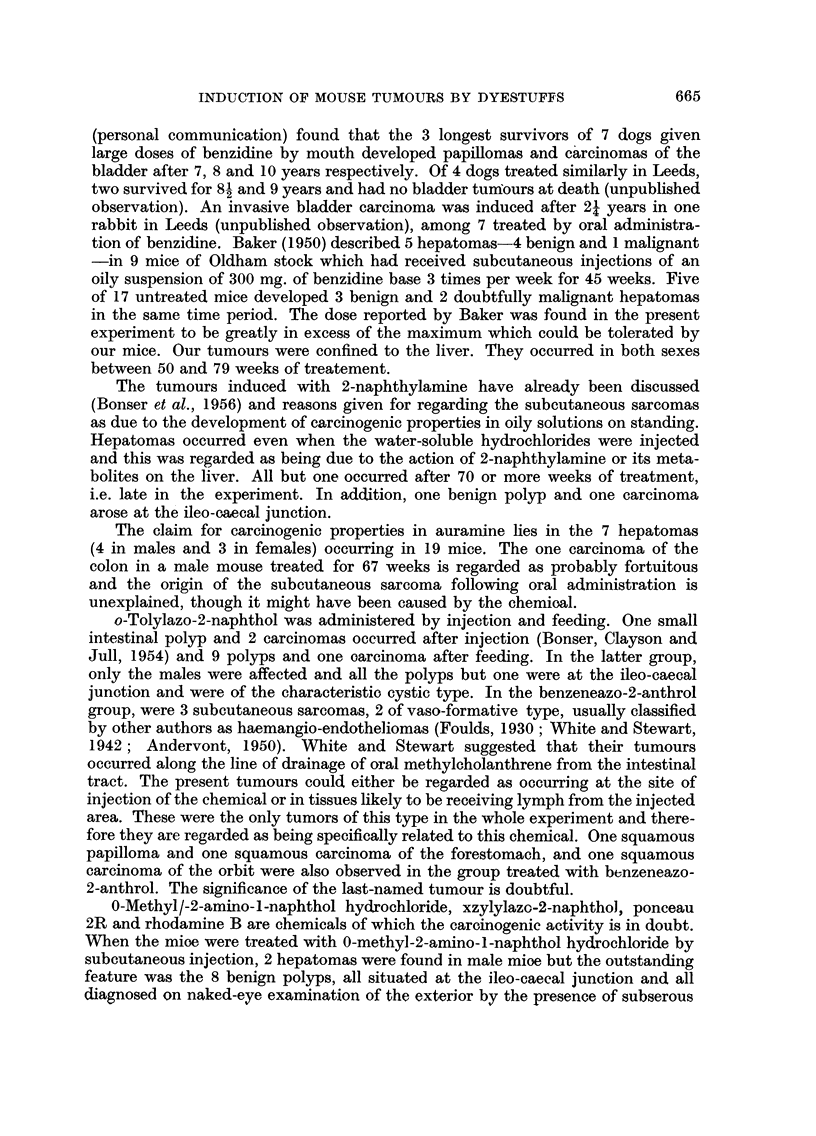

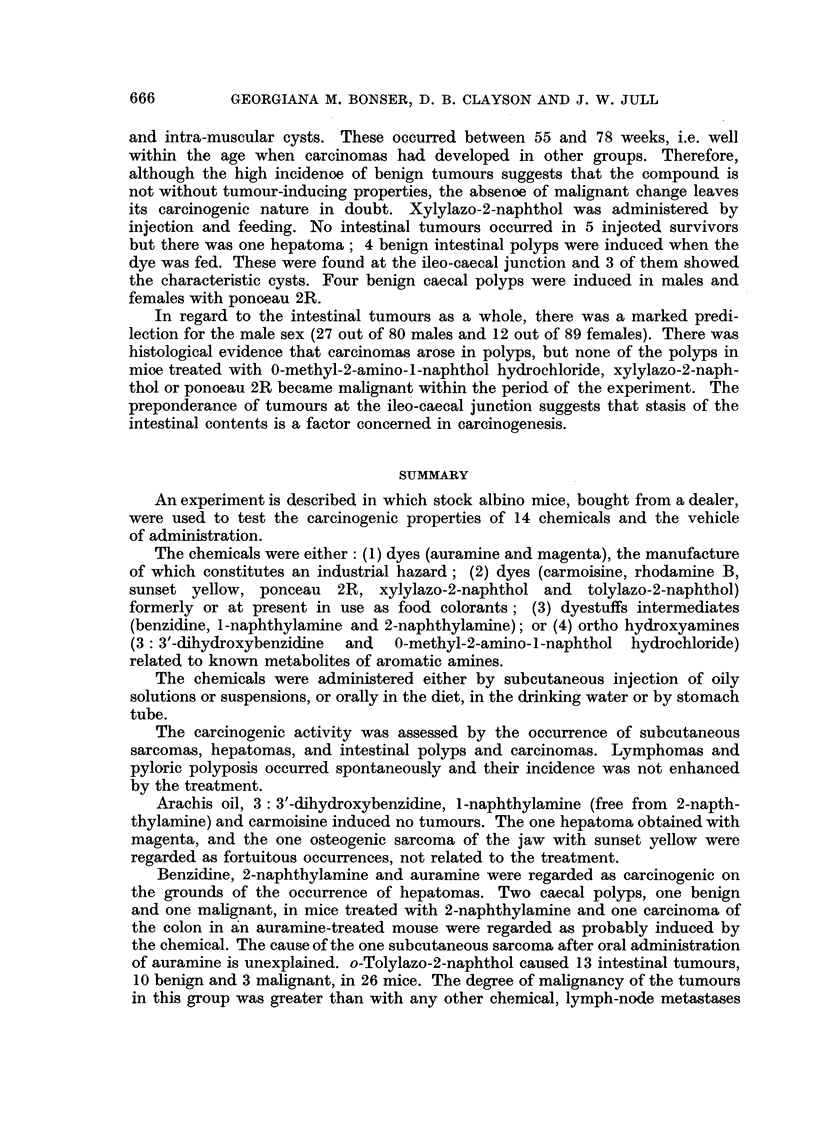

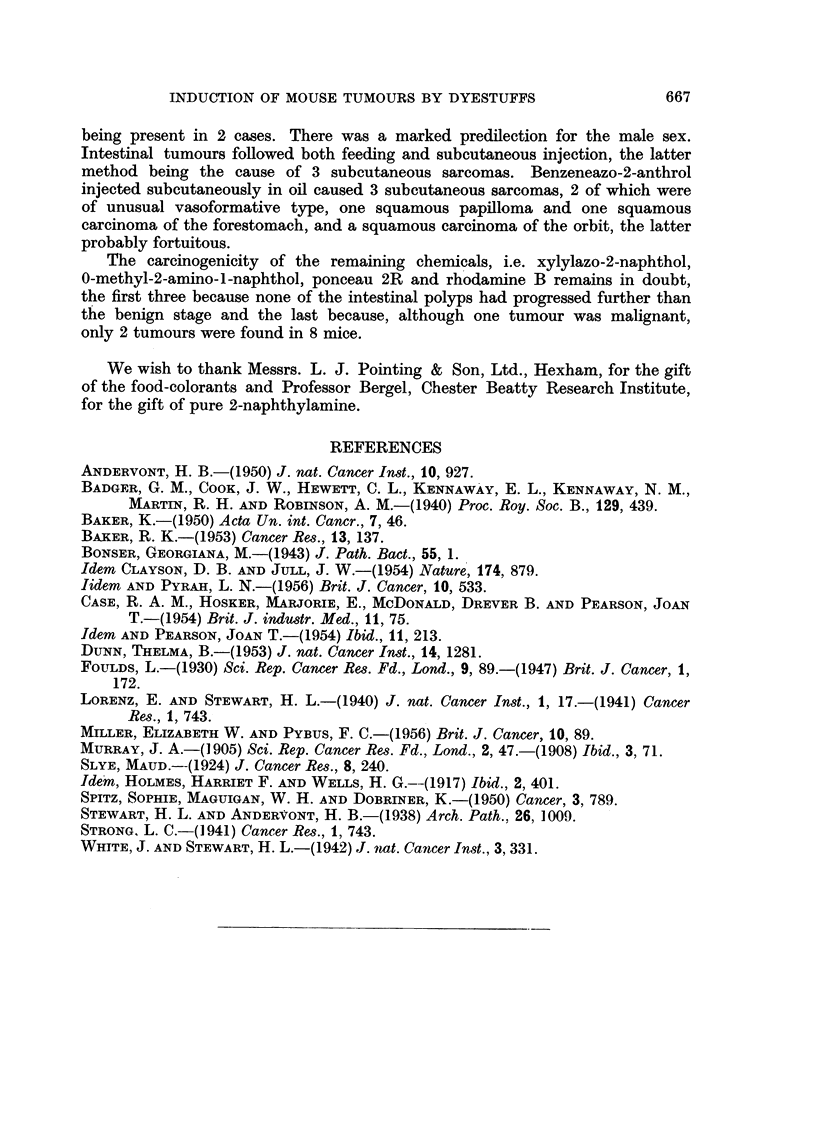


## References

[OCR_01436] ANDERVONT H. B. (1950). Induction of hemangioendotheliomas and sarcomas in mice with O-aminoazotoluene.. J Natl Cancer Inst.

[OCR_01445] BONSER G. M., CLAYSON D. B., JULL J. W. (1954). Induction of tumours with 1-(2-tolylazo)-2-naphthol (oil orange TX).. Nature.

[OCR_01470] SPITZ S., MAGUIGAN W. H., DOBRINER K. (1950). The carcinogenic action of benzidine.. Cancer.

